# Public Health Impact of Extremely Low-Frequency Electromagnetic Fields

**DOI:** 10.1289/ehp.8977

**Published:** 2006-06-22

**Authors:** Leeka Kheifets, Abdelmonem A. Afifi, Riti Shimkhada

**Affiliations:** 1 Department of Epidemiology and; 2 Department of Biostatistics, School of Public Health, University of California, Los Angeles, California USA

**Keywords:** attributable fraction, ELF, extremely low-frequency electromagnetic fields, health impact, health policy

## Abstract

**Introduction:**

The association between exposure to extremely low-frequency electric and magnetic fields (ELF) and childhood leukemia has led to the classification of magnetic fields by the International Agency for Research on Cancer as a “possible human carcinogen.” This association is regarded as the critical effect in risk assessment. Creating effective policy in light of widespread exposure and the undisputed value of safe, reliable, and economic electricity to society is difficult and requires estimates of the potential public health impact and associated uncertainties.

**Objectives:**

Although a causal relationship between magnetic fields and childhood leukemia has not been established, we present estimates of the possible pubic health impact using attributable fractions to provide a potentially useful input into policy analysis under different scenarios.

**Methods:**

Using ELF exposure distributions from various countries and dose–response functions from two pooled analyses, we calculate country-specific and worldwide estimates of attributable fractions (AFs) and attributable cases.

**Results:**

Even given a wide range of assumptions, we find that the AF remains < 10%, with point estimates ranging from < 1% to about 4%. For small countries with low exposure, the number of attributable cases is less than one extra case per year. Worldwide the range is from 100 to 2,400 cases possibly attributable to ELF exposure.

**Conclusion:**

The fraction of childhood leukemia cases possibly attributable to ELF exposure across the globe appears to be small. There remain, however, a number of uncertainties in these AF estimates, particularly in the exposure distributions.

There has been considerable scientific research to understand the potential link between residential and occupational exposures to power-frequency electric and magnetic fields (ELF) and the occurrence of cancer and other diseases. The “limited” association between childhood leukemia and ELF found in epidemiologic studies (evidence from epidemiologic studies is a major force in driving ELF risk assessments), along with “inadequate” animal data, has led to the classification of magnetic fields by the International Agency for Research on Cancer (IARC) as a “possible human carcinogen” (IARC 2002). Because childhood leukemia is the outcome for which the scientific evidence is strongest, it can be regarded as the critical effect in risk assessment and risk evaluation.

The combination of the association of childhood leukemia with low-level, chronic exposures on the one hand and the widespread exposures to ELF on the other have made it both necessary and difficult to develop consistent public health policies on ELF exposure. The need for such policies results from the undisputed value of safe, reliable, and economic electricity to society to maintain all the benefits that this provides. Creating effective policy in light of these critical considerations requires an estimate of the potential public health impact and associated uncertainties.

The attributable fraction (AF) can be used to inform policies on ELF exposure. The AF, based on an established exposure–disease relationship, is the proportion of the caseload (of disease) that is attributable to the exposure, assuming there is a causal relationship. Hence, the AF can be used to estimate the degree of incidence reduction that would be expected if the observed association were real and if exposure were reduced. In this article we begin with a description of what is known about ELF exposure distributions in various countries. Then, we calculate country-specific and worldwide estimates of AF and attributable cases based on dose–response functions of ELF and childhood leukemia from two major pooled analyses. We conclude with a discussion of the variability and uncertainty in these estimates. Although the causal relationship between magnetic fields and childhood leukemia has not been established (IARC 2002; Kheifets et al. 2005a, 2005b), we present estimates of the possible pubic health impact to provide potentially useful input into policy analysis.

## Methods

### Exposure distributions

When evaluating the risks from exposure to any biologically active agent—physical, biological, or chemical—it is important to understand the distribution and magnitude of the exposure in the general population. To effectively quantify the risks of childhood leukemia, if any, from exposure to ELF, we must first obtain some estimate of the degree of exposure in children. These exposures differ from country to country because of a number of factors, most notably the frequency and voltage used for power distribution, and population density. In the absence of a known or even plausible biophysical mechanism on which to base an etiologically relevant exposure measure, the exposure summary used in most epidemiologic studies has been the time-weighted average field. There are two types of studies from which the exposure distribution is extracted: *a*) exposure surveys to provide estimates of the exposure prevalence in the population (P0), and *b*) case series from case–control studies to provide estimates of P0 (prevalence in controls) and the exposure prevalence in children with childhood leukemia (P1). Each of these sources provides some advantages. Case–control studies provide most relevant measurements of exposure and focus on a 24-hr or longer measurement in the child’s bedroom. Estimates from case–control studies, however, might be biased if, for example, restrictions on the population (e.g., to live within a certain distance of power lines) make the case–exposure prevalence in the study different from the population prevalence; this bias renders unusable the case and control prevalence estimates from studies with exposure-related restrictions. Even if cases were representative of the population, the controls would not be representative if matching has been done and the matching factors are associated with exposure; in that case the P0 estimate from the study would be biased upward, toward P1. Fortunately, the most common matching factors were child’s age and sex, which appear to be almost independent of exposure in the studies (Greenland 2001a). Exposure surveys, on the other hand, typically include both children and adults, as well as personal measurements throughout the day, and thus are only tangentially related to the exposure in the child’s bedroom. At the very least the use of both of these sources provides a range of relevant exposures and subsequently a range of impact measures for consideration.

Five extensive surveys have been conducted to evaluate ELF exposures of the general population [Brix et al. 2001; Decat et al. 2005; Electric Power Research Institute (EPRI) 1993; EMF Rapid Program 1998; Yang et al. 2004]. These surveys generally estimate that approximately 4–5% had mean exposures > 0.3 μT with the exception of Korea where 7.8% had mean exposures > 0.3 μT. Only 1–2% has median exposures in excess of 0.4 μT. See Tables 1 and 2 for details of the exposure distributions used.

Estimating exposures using the case exposures from case–control studies allows us to look at a broader spectrum of countries and results in a range of 0.5–6.6% having mean exposures > 0.3 μT and 0.5–3.3% having median exposures > 0.4 μT. Two countries, the United States and Germany, had both exposure surveys and case–control studies. In the United States the mean exposures were virtually equal from the two methods, but the case–control median estimates were less than the survey median estimates. In Germany the case–control mean exposure estimates were substantially smaller than the survey estimates (median estimates were not available for the case–control study), which could be due to regional differences and the inclusion of occupational exposures in the survey estimates. In some studies the exposure distribution for 0.2–0.3 and 0.3–0.4 μT had to be estimated, as data were given only for the 0.2- to 0.4-μT intervals; the ratio from the U.S. Rapid Survey (EMF Rapid Program 1998; 7.8% for 0.2–0.3 μT and 2.4% for 0.3–4 μT) was used to calculate these estimates.

In addition to assuming no significant difference in the exposure distributions based on exposure surveys and case–control studies, we have assumed that exposures obtained using personal measures are equivalent to those from household measurements, regardless of the length of time of measurement and regardless of whether they were for children or adults. This last assumption was tested using average and geometric mean household measurements (bedroom and home) from the U.S. Rapid Survey (EMF Rapid Program 1998), which included a sample of both children and adults. A two-sample *t*-test with equal variances comparing the distributions of the log of the average measurements of total home exposure showed no significant difference between the distributions for children (< 15 years) and adults (≥ 15 years). Also, no significant difference was found between the adult and child distributions of the log of the geometric mean of total household exposure. The tests described above assumed that the log of the exposure is normally distributed, an assumption that we tested and found to fit the data well. Additional tests were conducted using either the Pearson chi-square or Fisher’s exact tests to compare the percent of people > 0.3 or 0.4 μT for children and adults; these tests showed no significant difference in the percent of children and adults above these exposure levels for total household ELF exposure.

### Dose response

Dose–response functions from two pooled analyses were used for estimating the risk ratios (RRs). In one pooled analysis based on nine well-conducted studies, virtually no excess risk was noted for exposure to ELF magnetic fields < 0.4 μT geometric mean exposure and a 2-fold excess risk was seen for exposure > 0.4 μT: The effect estimates and associated 95% confidence intervals (CIs) for all categories in relation to the lowest exposure category of < 0.1 μT are as follows: 1.08 (0.89–1.31) for exposures 0.1 μT to < 0.2 μT, 1.11 (0.84–1.47) for 0.2 μT to < 0.4 μT, and 2.00 (1.27–3.13) for exposures ≥ 0.4 μT (Ahlbom et al. 2000).

The other pooled analysis included 15 studies based on less restrictive inclusion criteria and used 0.3 μT arithmetic mean exposure as the highest cutpoint (Greenland et al. 2000). The effect estimates and associated 95% CI for all categories in relation to the lowest exposure category of ≤ 0.1 μT are as follows: 1.01 (0.84–1.21) for exposures > 0.1 μT to ≤ 0.2 μT, 1.06 (0.78–1.44) for > 0.2 μT to ≤ 0.3 μT, and 1.68 (1.23–2.31) for exposures > 0.3 μT (Greenland et al. 2000).

Overall, the two analyses are in close agreement, but one of the differences between the two pooled analyses is in the exposure metric used: Ahlbom et al. (2000) examined the association between the geometric mean ELF level and childhood leukemia in 9 epidemiologic studies, whereas Greenland et al. (2000) used the arithmetic mean to examine this association in 12 studies. The other difference between the analyses of Ahlbom et al. and Greenland et al. is the exposure categorization: < 0.1 μT, 0.1 to < 0.2 μT, 0.2 to < 0.4 μT, and ≥ 0.4 μT in Ahlbom et al. (2000), and ≤ 0.1 μT, > 0.1 to ≤ 0.2 μT, > 0.2 to ≤ 0.3 μT, and > 0.3 μT in Greenland et al. (2000). Greenland (2005) extended the analysis to include 14 studies using a cut point at 0.3 μT with similar results. Because most ELF exposure data are skewed, the log-normal distribution is often assumed as an approximation. In statistical analyses of such data, we usually take the log in order to equalize the variance among groups and to produce approximate normality, thereby conforming to the usual assumptions. Mathematically, the mean of the logged values is the log of the geometric mean. For this reason, a number of researchers use the geometric mean to summarize ELF exposure data. Alternatively, some authors argue that in a standard setting we need the risk at average exposure for a group to be equal to the average risk to that group (Crump 1998). This logic then leads to using the arithmetic mean to summarize the data. Because there are arguments for using both geometric and arithmetic means, we present two sets of AF estimates, one based on each type of mean.

### Attributable fraction

The AF is based on the counterfactual contrast between the number of cases in a population that occur when the population is subject to a given exposure distribution and the number that would occur in the same population if that distribution were changed (e.g., if exposure were reduced or eliminated by an intervention), assuming all other population characteristics remain the same. There are two basic pieces of information needed to make a crude estimate of the AF: an estimate of the exposure effect on the disease and the prevalence of exposure in the population. If no adjustment for covariates is needed, these values are simply substituted into the unadjusted (crude) AF formula (Levin 1953):


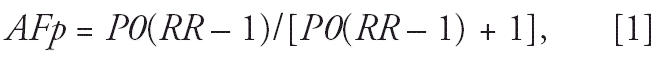


where *AFp* is the estimated AF, *RR* is the risk ratio estimate, and *P0* is the estimated exposure prevalence in the target population. In this article we use this formula to compute the AF for exposure survey studies.

For case–control studies with adjusted odds ratios (ORs), Levin (1953) gives another formula:


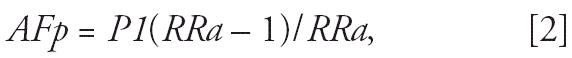


where *RRa* is the adjusted rate ratio estimate (study OR) and *P1* is the exposure prevalence among the cases in the target population (Rothman and Greenland 1998).

This formula has the advantages of requiring no adjustment of *P1* to be valid and is unaffected by matching controls to cases. We used both formulas, with minor differences in AF and report only the results based on the case–series exposure distribution (*P1*) here. To make these calculations for the ELF–childhood leukemia relation, as leukemia is a rare disease, we can assume that the OR estimates the RR. We must also assume that the risk ratio estimates the effect in the target population, that there is no bias, and that there is no change in the effect estimate moving from the study to the target population (Greenland 2004).

We also calculate the excess number of cases attributable to exposure, which was obtained by multiplying the AF by the total number of cases. We used the reported upper and lower confidence limits of the RR to compute upper and lower bounds of the estimated AF. It should be noted that the computed upper and lower bounds for the estimated AF holds only under the additional assumption that the exposure distribution in the population is known (or can be estimated).

### Attributable numbers

Leukemia is the most common childhood malignancy, constituting more than one-third of all childhood cancers. For children < 15 years of age, the estimated number of new leukemia cases in 2000 was approximately 49,000 globally, translating into an incidence rate of about 3 cases per 100,000 [International Association of Cancer (IACR) 2000]. See Table 3 for the global distribution of childhood leukemia incidence rates.

The number of cases attributable to EMF can be estimated by multiplying the AF by the total number of cases. The exposure distributions used to come up with country-specific AFs represent only a handful of countries across the world. We had exposure distributions, and hence AF estimates, for countries in North America, Europe, and Asia. To calculate a range of estimates for the attributable number (AN) for each continent, we used the lowest and highest estimates of AFs in each continent and multiplied each by the total number of leukemia cases in the continent to come up with a range of ANs. We used the corresponding CIs of the AFs to compute a derived 95% CI for the estimated number of cases. Where there were no studies from any representative country in the continent, such as Africa, Latin America, and Oceania, the overall lowest AF and highest AF estimates were used. Note that the Yang et al. (2004) study, which is based on a larger sample and considered more representative for non-Western regions, was used to calculate an upper range for regions with unknown levels (Latin America, Africa, Oceania).

### Exposure reduction scenario

The AN numbers for high exposures are the numbers of cases that would be averted if we were to eliminate exposures > 0.3 μT or 0.4 μT. However, realistically, it is difficult or impossible to determine locations in which such exposures exist and to eliminate them. An alternative approach might be an attempt to reduce exposures where possible to do so at no or low cost (Kheifets et al. 2005a).

To evaluate this approach, we estimated the impact of a hypothetical scenario where the population’s exposure distribution is reduced by 50%; in other words, each child receives half the exposure he or she was previously getting. A new exposure distribution was calculated to reflect this change. Because the empirical distribution of exposure measurements does not offer enough information to estimate a distribution shift, we created a calculated log-normal distribution based on the mean, standard deviation, and lowest observed value (0.01 μT). This type of detail was available for only one of the distributions (EMF Rapid Program 1998). Hence, our calculated distribution and estimates of exposure reduction are made only for the United States. To make the current and 50% reduction scenarios more comparable, we based both computations on the calculated, rather than the actual, log-normal distribution.

## Results

### Country-specific estimates

**Figure 1:**
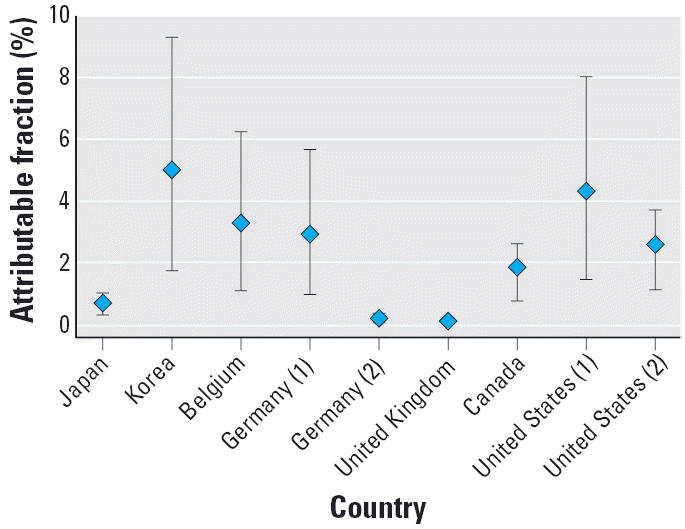
Upper, lower, and point estimates of AF, based on arithmetic mean exposure of ELF. Numbers in parentheses indicate multiple studies for one country. Figure is based on exposure distributions for specific countries and estimate of effect from pooled analysis by Greenland et al. (2000); Japan: Kabuto et al. (2006); Korea: Yang et al. (2004)); Belgium: Decat et al. (2005); Germany (1): Brix et al. (2001); Germany (2): Schuz et al. (2001); United Kingdom: UKCCS (1999); Canada: McBride et al. (1999); United States (1): EMF Rapid Program (1998); United States (2): Linet et al. (1997). Vertical bars indicate upper and lower AF estimates.

**Figure 2:**
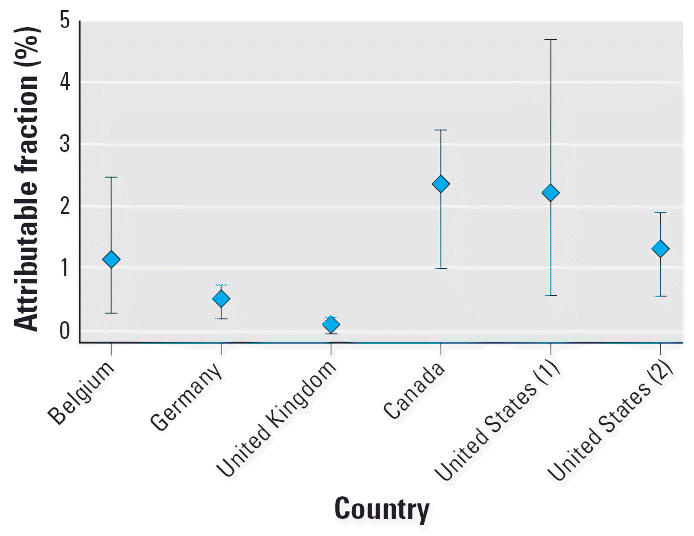
Upper, lower, and point estimates of AF, based on geometric mean exposure of ELF. Numbers in parentheses indicate multiple studies for one country. Figure is based on exposure distributions for specific countries and estimate of effect from pooled analysis by Ahlbom et al. (2000): Belgium: Decat et al. (2005); Germany: Michaelis et al. (1998); United Kingdom: UKCSS (1999); Canada: McBride et al. (1999); United States (1): EMF Rapid Program (1998); United States (2): Linet et al. (1997). Vertical bars indicate upper and lower AF estimates.

We computed point estimates, as well as upper and lower estimates of AFs for countries for which we have access to an exposure distribution (Figures 1, 2). For the United States and Germany, where there were multiple distributions, the largest of the case–control studies and the largest of the exposure surveys were used. Despite large differences in the information and assumptions from different studies and countries, the estimate of AFs remains low at around 3–4% and well below 1% for some countries.

### Potentially averted cases

AF and AN estimates for exposures > 0.1, 0.2, 0.3 or 0.4 μT were calculated for the arithmetic mean and geometric mean exposure distributions, before and after making the 50% exposure reduction in the United States (Table 4). The differences in the ANs reflect the numbers of cases that would be potentially averted in the United States because of the 50% exposure reduction. Interestingly, 50% reduction in the lower exposure categories (which is likely to be technically impossible and/or prohibitively expensive) results in the number of cases similar in magnitude to that which would be due to high exposure only.

### Worldwide estimates

**Figure 3:**
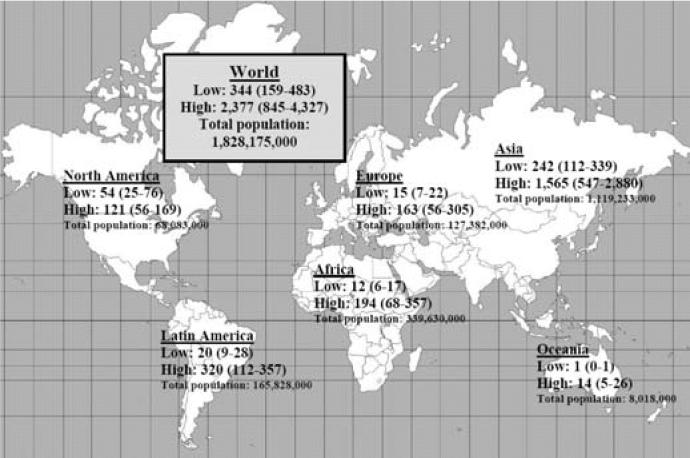
Estimated number and range of worldwide and regional cases of childhood leukemia among children < 14 years of age that are possibly attributable to EMF arithmetic mean exposure > 0.3 μT (and the corresponding derived 95% CI). Regional range is based on the lowest level and highest exposure levels from the countries in a given region. Where there was no information from any countries in the region, the overall lowest and highest exposure levels were used.

**Figure 4:**
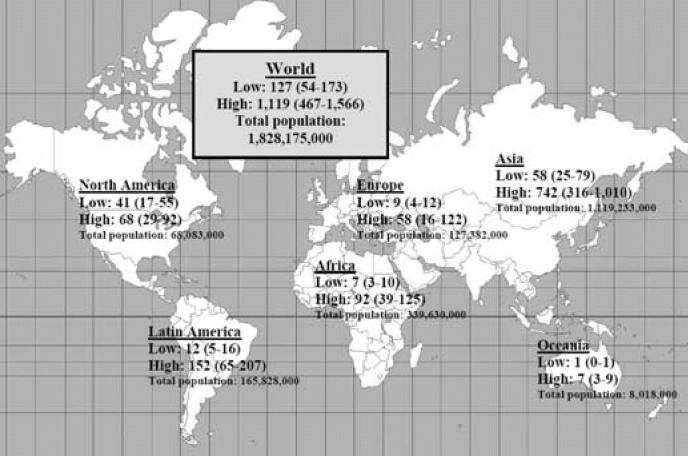
Estimated number and range of worldwide and regional cases of childhood leukemia among children < 14 years of age that are possibly attributable to EMF geometric mean exposure ≥ 0.4 μT (and the corresponding derived 95% CI). Regional range is based on the lowest level and highest exposure levels from the countries in a given region. Where there was no information from any countries in the region, the overall lowest and highest exposure levels were used.

The ANs of leukemia cases were calculated for the scenarios of eliminating arithmetic mean exposure > 0.3 μT and of eliminating geometric mean exposure ≥ 0.4 μT. This computation was made for regions around the world, then added to obtain a global estimate (Figures 3, 4). To compute these regional estimates, we used the lowest level and highest exposure levels estimated in Tables 1 and 2 from the countries in that region to obtain a regional range. Where no information was available from any countries in the region, we used the lowest and highest exposure levels overall. Making certain assumptions about possible exposure reduction scenarios, we provide a range of ANs thought to be most useful for policymaking. Reducing exposures to < 0.3 μT (arithmetic mean) or 0.4 μT (geometric mean) results in a number of averted cases (assuming causality) ranging from 100 to 2,400 cases annually worldwide.

## Discussion

Since the first report suggesting an association between residential ELF electric and magnetic fields and childhood leukemia was published in 1979 (Wertheimer and Leeper 1979), dozens of increasingly sophisticated epidemiologic studies have examined this association. In addition numerous comprehensive reviews, meta-analyses, and two pooled analyses have been published (e.g., Ahlbom et al. 2000; Greenland et al. 2000; IARC 2002; National Institute of Environmental Health Sciences 1999; National Research Council 1997). Making certain assumptions about possible exposure reduction scenarios, we provide a range of ANs thought to be most useful for policymaking. Globally, there is disproportionately more information on exposure from industrialized countries; there are a number of regions of the world, such as Africa and Latin America, where no representative information on exposure is available. Although the ORs from different study regions (when available) are similar, there are substantial differences in the exposure distributions even within countries. Comparable or larger differences are expected to exist elsewhere (e.g., exposures in China and India are probably very different from those in Korea). Therefore, the AF estimates cannot be confidently generalized to developing countries. Moreover, because these calculations are highly dependent on the exposure distribution and other assumptions, they are very imprecise. For small countries with low exposure, the number of attributable cases is less than one extra case per year. Worldwide the range is from 100 to 2,400 cases per year, possibly attributable to ELF exposure, representing a small proportion of total leukemia cases.

### Uncertainty

Random error has many components, including temporal and geographic variation, inter- and intraperson varariability, as well as errors in measurement. Intraperson, temporal, and geographic variability are usually accounted for by averaging measurements made via personal monitors and/or measurements made in several locations over a specified period of time; this is essential and many ELF studies include extensive measurements, often with frequent sampling during measurement periods. Methods are available to account for errors in measurement (Carrol et al. 1995), but all studies on ELF effects ignore this aspect. Consequently, these studies most likely underestimate the true degree of uncertainty of their conclusions (Greenland 2001b, 2003, 2005; Phillips 2003).

In childhood leukemia, both pooled analyses performed in 2000 were based on large numbers and hence resulted in RR estimates with tight confidence intervals. When analyses are compared, they demonstrate consistency in the size of their effect estimates and range of confidence intervals. It appears unlikely that random variability (or chance) played a significant role in the observed effect estimates of both pooled analyses. However, this does not exclude the possibility that exposure was assessed with a large degree of random error, which could bias the observed RR toward the null and introduce a lot of uncertainty into the potential dose response. All attempts to examine potential confounding have not changed the risk estimates and substantial confounding from factors that do not represent an aspect of the electric or magnetic fields is unlikely. Selection bias may be partially responsible for the consistently described epidemiologic association between ELF and childhood leukemia (Mezei and Kheifets 2005). A large study by Linet et al. (1997) drives the overall risk estimate in both pooled analyses and may have had the greatest potential for selection bias, thereby potentially inflating the risk estimate associated with EMF exposure. Therefore, our estimates of both AFs and ANs, which are based on the pooled analyses, may similarly be overestimated

### Conventional vs. Bayesian analysis

We present calculations of AFs that do not reflect any source of uncertainty other than random error and offer some informal judgments regarding the effect of possible biases. To provide additional input to policy analysis, Greenland and Kheifets (2006) employed formal Bayesian analyses to account for uncertainties about study biases as well as uncertainties about exposure distributions. They developed a model to account for the fraction attributable to exposure exceeding 0.3 μT and model the log of the counts of cases as a linear function of whether the measured ELF exposure and the unmeasured (unknown) true exposure is > 0.3 μT. Using data from the published literature, they formulated plausible prior distributions for exposure parameters, rate ratios, uncontrolled confounders, and for the degree of misclassification. They then combined this information to compute the posterior distribution of the fraction attributable to the true exposure. Their analyses support the idea that the public health impact of residential fields is likely to be limited, but the possibilities of both no impact and a large impact remain in light of the available data. The differences between their analyses and ours vary in both directions, but generally the Bayesian results produce much wider bounds than ours. Greenland and Kheifets (2006) argue that conventional analyses such as the ones presented here may be overoptimistic and over-confident. Nonetheless, we believe that both the conventional and Bayesian approaches offer policy analysts additional insights that should be helpful in disentangling the uncertainties inherent in this field. Using the simpler conventional methods in this article, we explore a variety of cut points, evaluate exposure reduction scenarios, and calculate worldwide estimates.

## Conclusion

At present it is not possible to decide whether ELF exposure increases the risk of childhood leukemia, which is reflected in the IARC classification of magnetic fields as a “possible” carcinogen. To estimate the potential public health impact, we have calculated a range of AFs under different scenarios. Even given a wide range of assumptions, the AF remains low, with point estimates ranging from < 1% to about 4%. As the AF is highly dependent on the exposure distribution, more data are needed on exposure levels worldwide, which should be collected in a large systematic survey of an appropriately selected sample.

## Figures and Tables

**Table 1:** Exposure distributions of arithmetic mean (μT) ELF measurements.^a^

				Magnetic field category (μT)				
Country	Study	Study type	Measurement	≤ 0.1 (%)	> 0.1 – ≤ 0.2 (%)	> 0.2 – ≤ 0.3 (%)	> 0.3 (%)	*n*
Belgium	Decat et al. 2005	Exposure survey	24-hr personal	81.9	11.5	1.6	5.1	251
Canada	McBride et al. 1999^b^	Case–control	48-hr personal	58.59	25.93	10.77	4.71	297
Germany	Michaelis et al. 1998^b^	Case–control	24-hr bedroom	85.23	9.66	1.70	3.	176
	Brix et al. 2001	Exposure survey	24-hr personal	73.6	17.8	4.1	4.	1,952
	Schuz et al. 2001^c^	Case–control	24-hr bedroom	91.83	6.42	0.97	0.7	514
Japan	Kabuto et al. 2006^c^	Case–control	7-day home	88.46	5.77	3.85	1.92	312
Korea	Yang et al. 2004	Exposure survey	24-hr personal	64.0	24.2	4.0	7.8	409
United Kingdom	UKCCS 1999^c^	Case–control	48-hr home	92.73	5.31	1.49	0.47	1,073
United States	London et al. 1991^b^	Case–control	24-hr bedroom	67.90	18.52	3.09	10.49	162
	Linet et al. 1997^b^	Case–control	24-hr bedroom	63.17	23.82	6.43	6.58	638
	EMF Rapid Program 1998	Exposure survey	24-hr personal	64.2	21.1	7.8	6.6	995
	EPRI 1993	Exposure survey	24-hr home	72.3	17.5	5.6	4.6	987

UKCCS, UK Childhood Cancer Study.

a Based on exposure of cases in case–control studies and all respondents in exposure surveys.

b Based on Greenland et al. (2000) reported distribution for pooled analysis.

c Exposure categories: < 0.1, 0.1 to < 0.2, 0.2 to < 0.4, ≥ 0.4.

**Table 2:** Exposure distributions of geometric mean (μT) ELF measurements.^a^

				Magnetic field category (μT)				
Country	Study	Study type	Measurement	< 0.1 (%)	0.1 – < 0.2 (%)	0.2 – < 0.4 (%)	≥ 0.4 (%)	*n*
Belgium	Decat 2005	Exposure survey	24-hr personal	91.9	4.1	2.8	1.2	251
Canada	McBride et al. 1999^b^	Case-control	48-hr personal	63.97	20.59	10.66	4.78	272
Germany	Michaelis et al. 1998^b^	Case-control	24-hr bedroom	89.14	6.86	2.86	1.14	175
United Kingdom	UKCSS 1999^b^	Case-control	48-hr home	94.87	3.54	1.21	0.37	1,073
United States	EMF Rapid Program 1998^b^	Exposure survey	24-hr personal	72.6	17.6	7.5	2.3	995
	Linet et al. 1997^b^	Case-control	24-hr bedroom	70.25	18.66	8.24	2.86	595

a Based on exposure of cases in case–control studies and all respondents in exposure surveys.

b Based on Ahlbom et al. (2000) reported distribution for pooled analysis.

**Table 3:** Global incidence of childhood leukemia for children < 14 years of age in 2000.

		Childhood leukemia	
Region	Population of 0- to 14 year olds^a^	New cases^b^	Incidence rate (per 100,000)
Africa	339,631,000	3,848	1.13
Asia	1,119,233,000	31,062	2.78
Europe	127,382,000	4,878	3.83
Latin America	165,828,000	6,367	3.84
North America	68,083,000	2,841	4.17
Oceania	8,018,000	283	3.53
World	1,828,175,000	49,000	2.68

a Data from IACR (2000).

b Data from United Nations (2002).

**Table 4:** Point and low and high estimates (in parentheses) of the proportion (AF) and number (AN) of cases in the United States for the hypothetical scenario of 50% reduction in exposure of ELF.

	Exposures above:		
Arithmetic mean	0.1 μT	0.2 μT	0.3 μT
Proportion of all cases attributable to exposure (AF): current exposure distribution^a^	5.41% (−3.78%, 16.48%)	5.18% (−0.05%, 11.96%)	4.73% (1.65%, 8.73%)
Hypothetical distribution:^b^ all exposures decreased by 50%	1.27% (−2.02%, 5.29%)	1.16% (−0.21%, 3.02%)	1.01% (0.34%, 1.93%)
Number of cases attributable to exposure (AN): current exposure distribution	138 (−97, 421)	133 (−1, 306)	121 (42, 223)
Hypothetical distribution: all exposures reduced by 50%	32 (−52, 135)	30 (−5, 77)	26 (9, 49)
Number of cases averted due to exposure reduction	105 (−45, 286)	103 (4, 228)	95 (33, 174)

	Exposures above:		
Geometric mean	0.1 μT	0.2 μT	0.4 μT
Proportion of all cases attributable to exposure (AF): current exposure distribution^a^	3.95% (−2.83%, 12.30%)	2.46% (−0.71%, 6.77%)	1.67% (0.46%, 3.49%)
Hypothetical distribution:^b^ all exposures decreased by 50%	0.94% (−0.99%, 3.32%)	0.37% (−0.20%, 1.17%)	0.20% (0.05%, 0.42%)
Number of cases attributable to exposure (AN): current exposure distribution	101 (−72, 315)	63 (−18, 173)	43 (12, 89)
Hypothetical distribution: all exposures decreased by 50%	24 (−25, 85)	10 (−5, 30)	5 (1, 6)
Number of cases averted due to exposure reduction	77 (−47, 230)	53 (−13, 143)	38 (10, 83)

a Calculated log-normal distribution based on the EMF Rapid Program (1998).

b Calculated log-normal distribution based on the EMF Rapid Program (1998), with all exposures reduced by 50%.

## References

[b1-ehp0114-001532] Ahlbom A, Day N, Feychting M, Roman E, Skinner J, Dockerty J (2000). A pooled analysis of magnetic fields and childhood leukaemia. Br J Cancer.

[b2-ehp0114-001532] Brix J, Wettemann H, Scheel O, Feiner F, Matthes R (2001). Measurement of the individual exposure to 50 and 16 2/3 Hz magnetic fields within the Bavarian population. Bioelectromagnetics.

[b3-ehp0114-001532] Carrol RJ Rupert D Stefansi LA 1995. Measurement Error in Nonlinear Models. New York:Chapman & Hall.

[b4-ehp0114-001532] Crump KS (1998). On summarizing group exposures in risk assessment: is an arithmetic mean or a geometric mean more appropriate?. Risk Anal.

[b5-ehp0114-001532] Decat G Van den Heuvel I Mulpas L 2005. Final Report of the BBEMG Research Contract: June 10, 2005. Erembodegem, Belgium:Flemish Environmental Agency.

[b6-ehp0114-001532] Electric Power Research Institute (EPRI) 1993. Survey of Residential Field Magnetic Field Sources: September 1993. Palo Alto, CA:Electrical Power Research Institute.

[b7-ehp0114-001532] EMF Rapid Program 1998. Survey of Personal Magnetic Field Exposure, Phase II: 1000-Person Survey. Research Triangle Park, NC:National Institute of Environmental Health Sciences.

[b8-ehp0114-001532] Greenland S (2001a). Estimation of population attributable fractions from fitted incidence ratios and exposure survey data, with an application to electromagnetic fields and childhood leukemia. Biometrics.

[b9-ehp0114-001532] Greenland S (2001b). Sensitivity analysis, Monte Carlo risk analysis, and Bayesian uncertainty assessment. Risk Anal.

[b10-ehp0114-001532] Greenland S (2003). The impact of prior distributions for uncontrolled confounding and response bias: a case study of the relation of wire codes and magnetic fields to childhood leukemia. J Am Stat Assoc.

[b11-ehp0114-001532] Greenland S (2004). Model-based estimation of relative risks and other epidemiologic measures in studies of common outcomes and in case-control studies. Am J Epidemiol.

[b12-ehp0114-001532] Greenland S (2005). Multiple-bias modelling for analysis of observational data (with discussion). J R Stat Soc [Ser A].

[b13-ehp0114-001532] Greenland S, Kheifets L (2006). Leukemia attributable to residential magnetic fields: results from analyses allowing for study biases. Risk Anal.

[b14-ehp0114-001532] Greenland S, Sheppard A, Kaune W, Poole C, Kelsh M (2000). A pooled analysis of magnetic fields, wire codes, and childhood leukemia. Epidemiology.

[b15-ehp0114-001532] IACR (International Association of Cancer Registries) 2000. Globocan 2000 Database, Version 1.0. Available: http://www.iacr.fr [accessed 1 March 2005].

[b16-ehp0114-001532] IARC 2002. Non-Ionizing Radiation, Part 1: Static and Extremely Low-Frequency (ELF) Electric and Magnetic Fields. Lyon, France:International Agency for Research on Cancer. PMC5098132 12071196

[b17-ehp0114-001532] Kabuto M, Nitta H, Yamamoto S, Yamaguchi N, Akiba S, Honda Y (2006). Childhood leukemia and magnetic fields in Japan: a case-control study of childhood leukemia and residential power-frequency magnetic fields in Japan. Int J Cancer.

[b18-ehp0114-001532] Kheifets L, Repacholi M, Saunders R, van Deventer E (2005a). Sensitivity of children to EMF. Pediatrics.

[b19-ehp0114-001532] Kheifets L, Sahl JD, Shimkhada R, Repacholi MH (2005b). Developing policy in the face of scientific uncertainty: interpreting 0.3 microT or 0.4 microT cutpoints from EMF epidemiologic studies. Risk Anal.

[b20-ehp0114-001532] Levin ML (1953). The occurrence of lung cancer in man. Acta Unio Internationalis Contra Cancrum.

[b21-ehp0114-001532] Linet M, Hatch E, Kleinermann R, Robison L, Kaune W, Friedman D (1997). Residential exposure to magnetic fields and acute lymphoblastic leukemia in children. N Engl J Med.

[b22-ehp0114-001532] London S, Thomas D, Bowman J, Sobel E, Cheng T-C, Peters J (1991). Exposure to residential electric and magnetic fields and risk of childhood leukemia. Am J Epidemiol.

[b23-ehp0114-001532] McBride M, Gallagher R, Thériault H, Armstrong B, Tamaro S, Spinelli J (1999). Power-frequency electric and magnetic fields and risk of childhood leukemia in Canada. Am J Epidemiol.

[b24-ehp0114-001532] Mezei G, Kheifets L (2005). Selection bias and its implications for case-control studies: a case study of magnetic field exposure and childhood leukemia. Int J Epidemiol.

[b25-ehp0114-001532] Michaelis J, Schüz J, Meinert R, Zemann E, Grigat JP, Kaatsch P (1998). Combined risk estimates for two German population-based case-control studies on residential magnetic fields and childhood leukemia. Epidemiology.

[b26-ehp0114-001532] National Institute of Environmental Health Sciences 1999. NIEHS Report on Health Effects from Exposure to Power-Line Frequency Electric and Magnetic Fields. NIH Publ no. 99-4493. Research Triangle Park, NC:National Institute of Environmental Health Sciences.

[b27-ehp0114-001532] National Research Council 1997. Possible Health Effects of Exposure to Residential Electric and Magnetic Field. Washington, DC:National Academy Press. 25121270

[b28-ehp0114-001532] Phillips CV (2003). Quantifying and reporting uncertainty from systematic errors. Epidemiology.

[b29-ehp0114-001532] Rothman KJ Greenland S eds. 1998. Modern Epidemiology. Philadelphia:Lippincott-Raven.

[b30-ehp0114-001532] Schuz J, Grigat J, Brinkmann K, Michaelis J (2001). Residential magnetic fields as a risk factor for childhood acute leukaemia: results from a German population-based case-control study. Int J Cancer.

[b31-ehp0114-001532] UKCCS (UK Childhood Cancer Study) (1999). Exposure to power-frequency magnetic fields and the risk of childhood cancer. Lancet.

[b32-ehp0114-001532] United Nations 2002. World Population Prospects: The 2002 Revision Population Database. Available: http://esa.un.org/unpp/index.asp?panel=2 [accessed 1 March 2005].

[b33-ehp0114-001532] Wertheimer N, Leeper E (1979). Electrical wiring configurations and childhood cancer. Am J Epidemiol.

[b34-ehp0114-001532] Yang KH Ju MN Myung SH 2004. Sample of Korean’s occupational and residential exposures to ELF magnetic field over a 24-hour period. In: Abstracts of 26th Annual Meeting of the Bioelectromagnetics Society, 21–24 June 2004, Washington, DC. Washington, DC:Bioelectromagnetics Society, 188–189.

